# University of Edinburgh

**Published:** 1856-01

**Authors:** 


*University of Edinburgh*—Dr. Laycock.—We understand that Dr. Laycock has become the purchaser of the collection of pathological drawings belonging to the celebrated Dr. John Thompson, late Professor of Pathology in this University, and his son Dr. W. Thomson, late Professor of the Practice of Physic in the University of Glasgow. This collection consists of about 2500 delineations, more than one-half of which are original, unpublished colored representations of the morbid appearances and structures observed in various diseases. Nearly one-third of these were executed by the now Sir Robert Carswell, physician to the King of the Belgians; some from cases in this country; but the majority from cases occurring in the great hospitals of Paris, Lyons, Rome, and other continental cities, to which Mr. Carswell was sent by Dr. Thomson, accompanied by Dr. William Thomson. The remainder are by various artists of great experience and ability in the delineation of disease—such as Patrick Syme, Schethey McArtney, &c. The whole forms, we believe, the most valuable and extensive collection of pathological drawings in the world, and we are glad that this collection has once more returned to our University; and as it will be of immense advantage to several of our professors in the illustration of their courses, we hope that the Professors and Town Council, in the administration of the Reid Fund, will take care that this collection now becomes the property of the University, and not of any one professor. We can conceive no better application of part of the interest of this fund than to such a purpose.—*Daily Scotsman.*

